# Clean-Label Strategies for the Replacement of Nitrite, Ascorbate, and Phosphate in Meat Products: A Review

**DOI:** 10.3390/foods14142442

**Published:** 2025-07-11

**Authors:** Minhyeong Kim, Su Min Bae, Yeongmi Yoo, Jibin Park, Jong Youn Jeong

**Affiliations:** 1 Department of Food Science & Biotechnology, Kyungsung University, Busan 48434, Republic of Korea; pleomax159@naver.com (M.K.); smbae219@gmail.com (S.M.B.); yooym9964@naver.com (Y.Y.); ppasd444@naver.com (J.P.); 2 Brain Busan 21 Plus Project Team, Kyungsung University, Busan 48434, Republic of Korea; 3 Food & Life Science Research Institute, Kyungsung University, Busan 48434, Republic of Korea

**Keywords:** clean-label meat products, nitrite replacement, curing accelerator, phosphate replacers, plant-based ingredients

## Abstract

The clean-label movement has markedly increased consumer demand for meat products free from synthetic additives, such as sodium nitrite, ascorbate, and phosphate. This review summarizes strategies to replace these additives with natural alternatives while preserving the functional and quality properties of traditionally cured meats. Nitrite replacement commonly employs nitrate-rich vegetables, alongside nitrate-reducing starter cultures or pre-converted nitrite powders for adequate nitric oxide production and meat pigment stabilization. Ascorbate substitutes include vitamin C-rich materials and polyphenol-based antioxidants from green tea and rosemary, supporting nitrite reduction and contributing to meat pigment and oxidative stability. To compensate for phosphate functions, natural substitutes such as hydrocolloids, dietary fibers, protein isolates, and calcium powders from eggshells or oyster shells have shown partial success in restoring water-holding capacity, pH buffering, and textural integrity. In addition, non-thermal processing technologies, such as high-pressure processing, ultrasound, and cold plasma are explored as complementary strategies to enhance the efficacy of natural ingredients and support industrial scalability. However, challenges persist regarding ingredient variability, dose-dependent effects, and consistency in functional performance. Future research should focus on synergistic ingredient combinations, formulation standardization, and scalable application in industrial production to ensure the production of high-quality clean-label meat products.

## 1. Introduction

The increasing demand for clean-label meat products is primarily driven by heightened consumer awareness of food safety, ingredient transparency, and health-conscious choices. The term “clean label” generally refers to products made with a simplified list of natural, familiar, and minimally processed ingredients and without artificial additives, synthetic preservatives, or chemical-sounding names [1,2,3]. In this context, synthetic additives, such as sodium nitrite, ascorbate (or erythorbate), and phosphate, are under increased scrutiny due to their chemically derived names, potential health implications, and declining consumer acceptance [1,2]. These additives serve critical roles in meat processing: nitrite inhibits microbial growth and promotes the formation of nitrosyl hemochrome, a heat-stable pigment responsible for the characteristic pink color of cured meat; ascorbate converts nitrite to nitric oxide and prevents lipid oxidation; and phosphate enhances water-holding capacity (WHC), textural integrity, and emulsion stability [3,4,5]. However, growing concerns over N-nitroso compound formation linked to nitrite use, as well as metabolic health risks from excessive phosphate intake, have driven intensified reformulation efforts for developing safer and more transparent alternatives [6,7].

To address these concerns, various plant-derived alternatives have been extensively investigated. For nitrite replacement, nitrate-rich vegetables such as celery, beetroot, and radish, which are often combined with nitrate-reducing starter cultures or used in fermented, pre-converted forms, have shown potential as indirect sources of nitrite [8,9]. Similarly, natural curing accelerators, including vitamin C-rich ingredients (e.g., acerola and citrus peel) and polyphenol-rich antioxidants (e.g., rosemary and green tea), have emerged as viable alternatives to synthetic ascorbate owing to their reducing and antioxidative properties [10,11]. In phosphate-free systems, various functional substitutes, such as hydrocolloids, dietary fibers, protein-based binders, and naturally derived calcium powders, have shown partial effectiveness in restoring WHC, buffering ability, and textural attributes [12,13].

Despite these advances, such alternatives frequently encounter challenges in achieving the full functional role of traditional additive systems. The performance of natural substitutes is influenced by factors such as ingredient source, dosage levels, processing conditions, and interactions within the meat matrix, leading to variabilities in curing efficiency, color retention, oxidative stability, and sensory attributes [11,14,15]. Although numerous studies have proposed clean-label alternatives to individual additives, relatively few have systematically addressed the interdependent roles of nitrite, curing accelerators, and phosphate within integrated meat product systems [12,13,16,17,18]. Moreover, challenges remain regarding industrial-scale validation, process optimization, and economic feasibility. The ambiguity surrounding terms such as “natural curing”, “pre-converted nitrite”, and “nitrite-free” complicates labeling practices and may influence consumer perception [1,19]. It is important to recognize that the regulatory classification of certain natural ingredients, particularly those used for curing or preservation, does not uniformly align with clean-label standards across regulatory frameworks in different countries. These divergences reflect underlying differences in legislative definitions and consumer perception and underscore the necessity of region-specific considerations when evaluating and implementing clean-label strategies in meat products [3,16,20,21,22].

This review provides a critical evaluation of current clean-label strategies for replacing nitrite, curing accelerators such as ascorbate, and phosphate in meat products. Natural alternatives are systematically categorized by functional role, and their limitations and research needs are discussed. Emphasis is placed on synergistic ingredient systems and a unified framework that integrates both ingredient-based and technological approaches. Additionally, non-thermal processing technologies are examined as complementary strategies to improve functionality and support commercial application.

## 2. Clean-Label Strategies for Replacing Synthetic Additives in Meat Products

The shift toward clean-label meat products necessitates the replacement of three major synthetic additives: sodium nitrite, ascorbate (and its stereoisomer erythorbate), and phosphate. These compounds play essential roles in microbial inhibition, pigment formation, antioxidation, and water retention and are increasingly scrutinized by both consumers and regulatory authorities [2,3,4,5,16,23]. Consequently, the development of effective natural alternatives requires a comprehensive understanding of their chemical functionalities and the ability to achieve comparable effects using naturally derived compounds.

Sodium nitrite is often replaced with nitrate-rich vegetables, such as celery, beetroot, and spinach, which are typically available in powder or extract forms [24,25]. These vegetable-derived nitrate sources can be fermented or combined with nitrate-reducing starter cultures to generate nitrite in situ during processing [9,26,27]. Several studies have demonstrated that pre-converted vegetable powders and natural reductants can effectively facilitate cured color formation and inhibit lipid oxidation in nitrite-free systems [8,18,24]. However, achieving consistent nitrite conversion, color stability, and microbial safety remains challenging. To address these limitations, secondary components, such as fruit-derived polyphenols or plant-based reductants are often incorporated.

In the pursuit of replacing synthetic curing accelerators, naturally derived antioxidants, such as acerola, citrus peel extracts, green tea, rosemary, and grape seed extract, have demonstrated promising functionality [10,11,18,28]. These alternatives exhibit both reducing activity and antioxidative properties and are frequently combined with nitrate-rich vegetable powders to enhance curing reactions and stabilize cured pigments [18,28,29,30,31]. However, their effectiveness depends on the extraction method, ingredient compatibility, and stability under processing and storage conditions.

Phosphates, on the other hand, play essential roles in adjusting pH, improving protein solubility, and enhancing WHC and emulsion stability, which are crucial functions for maintaining product yield and textural attributes [4,5]. They are increasingly being replaced by dietary fibers (e.g., citrus and bamboo fibers), protein-based binders (e.g., soy or pea protein), and calcium powders derived from eggshell or seashell sources [5]. Furthermore, composite ingredient systems that integrate multiple clean-label components have demonstrated synergistic effects in replacing phosphate functionality [12,32].

**Figure 1:**
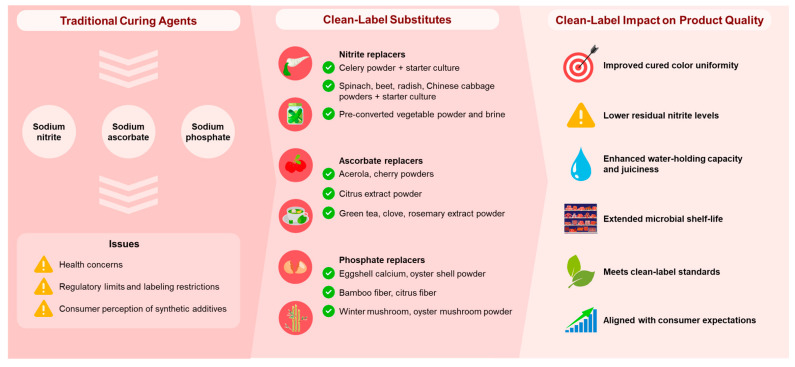
Strategic framework for replacing nitrite, ascorbate, and phosphate in clean-label meat products and their impact on key quality attributes.

Despite these advances, natural replacements often fall short of matching the multifunctional effects of conventional additives. Recent approaches therefore emphasize synergistic combinations and careful formulation to improve consistency, efficacy, and product quality. Figure 1 provides a strategic framework that summarizes the major ingredient categories used to replace nitrite, ascorbate, and phosphate in clean-label meat products and their associated effects on product quality.

### 2.1. Nitrite Replacement Strategies

Nitrite plays a pivotal role in cured meat products by stabilizing their pink color through the formation of nitrosyl hemochrome, providing antioxidative protection, and inhibiting pathogens, such as *Clostridium botulinum* [3,23,26]. Although plant-based nitrate combined with starter cultures has emerged as a common strategy in natural curing, it should be noted that the nitric oxide generated, regardless of its source, remains a potential precursor to carcinogenic N-nitroso compounds. Therefore, these systems do not inherently reduce toxicological risk [3,16]. Their growing adoption is primarily driven by clean-label positioning, regulatory allowances, and consumer preference for natural ingredients.

A primary clean-label curing strategy involves replacing synthetic nitrites with nitrate-rich vegetables, particularly celery powder, which is the most widely used in commercial meat production. Other vegetables, such as spinach, radish, beetroot, and Chinese cabbage, have also demonstrated nitrate levels and curing efficacy comparable to those of celery [33,34,35,36,37,38]. These vegetables are typically applied in their powder or extract form and contain high levels of naturally occurring nitrate, which can be microbially reduced to nitrite during processing. When combined with nitrate-reducing bacteria such as *Staphylococcus carnosus* under controlled pH, temperature, and salt conditions, this in situ conversion effectively reproduces traditional curing processes and is suitable for both cooked and dry-cured meat products [8,37,38,39,40,41].

Unlike in situ systems, pre-converted powders are generated through controlled fermentation processes, yielding stable nitrite concentrations suitable for direct incorporation. Pre-converted (fermented) vegetable powders, such as those derived from celery and Chinese cabbage, have been shown to facilitate cured pigment formation and enhance the oxidative stability of naturally cured products [18,42,43]. A similar principle applies to nitrite-containing brine systems, wherein vegetable extracts undergo fermentation in liquid form. These pre-fermented brines can promote uniform distribution and potentially enhance curing reactions under controlled conditions [35,44,45].

Vegetables with high nitrate content, such as celery, spinach, and radish, have been investigated for their potential to provide natural nitrate in alternative meat curing systems. Table 1 presents a summary of the representative nitrate-rich vegetables relevant to natural curing applications, detailing their nitrate concentrations and botanical families.

#### 2.1.1. Nitrate-Rich Vegetables for Natural Curing Applications

Nitrate-rich vegetables offer a fundamental strategy for substituting synthetic nitrites in clean-label meat products. Such vegetables serve as nitrate donors in natural curing systems, with their functional application depending on their nitrate content and compatibility with microbial or pre-converted systems [45,54], as discussed in subsequent sections.

Celery is a nitrate-rich vegetable that has been extensively studied and widely employed in natural curing systems, particularly in the form of standardized commercial powders, and is generally regarded as the industry benchmark. However, alternative nitrate-accumulating vegetables such as Chinese cabbage, radish, and spinach have also shown promising potential under optimized cultivation and processing conditions [26,34,36,37,45,55]. However, celery is also a recognized allergen in some regions (e.g., European Union countries), and its use requires careful labeling and allergen management to ensure consumer safety in clean-label applications [56].

Beyond celery, vegetables such as spinach, radish, Chinese cabbage, Swiss chard, and lettuce have been identified as significant nitrate accumulators, particularly under specific cultivation and environmental conditions [4,33,34,35,36,38,47,48,52,53]. These vegetables have also been investigated for their functional applications in various clean-label meat products [8].

As summarized in Table 1, the nitrate content of these vegetables varies considerably across different species and cultivation regions. The reported concentrations cover a broad spectrum, with spinach and radish identified as particularly nitrate rich in specific regional studies [4,34,47,48,50,52,53]. This variation is largely influenced by extrinsic factors such as soil nitrogen availability, fertilization regimes, and sunlight exposure [8,47,57,58,59]. Although members of the *Brassicaceae*, *Chenopodiaceae*, and *Apiaceae* families tend to accumulate nitrate and exhibit agronomic adaptability, the inherent variability in nitrate content poses challenges for achieving consistent functional outcomes in cured meat products [16,58,59,60]. Fluctuating nitrate levels caused by both genetic and environmental factors can result in unpredictable residual nitrite concentrations, thereby complicating product standardization, safety assurance, and regulatory compliance [35,47,57]. This highlights the importance of rigorous quality control of raw materials in the production of naturally cured products.

To effectively use these vegetables in natural curing applications, reliable nitrate quantification and pairing with suitable microbial systems are essential. However, their curing performance may vary depending on the processing conditions, requiring further evaluation to ensure consistency and efficacy.

#### 2.1.2. Nitrate Conversion System Using Starter Cultures

Building on the use of nitrate-rich vegetables introduced in Section 2.1.1, this section focuses on microbial systems that convert plant-derived nitrate into nitrite within meat products. Starter cultures are employed owing to their high nitrate reductase activity, which promotes the in situ reduction of nitrate to nitrite and facilitates nitric oxide generation. Nitric oxide subsequently binds to heme proteins to form nitrosyl hemochrome upon cooking, resulting in the characteristic cured color while contributing to microbial inhibition and lipid oxidative stability [4,8,45,54,61,62,63]. Effective implementation of these strategies requires selecting starter cultures capable of efficiently reducing vegetable-derived nitrates to nitrites under typical meat-processing conditions. Starter cultures such as *S. carnosus* and *Staphylococcus xylosus* are frequently employed owing to their robust nitrate reductase activity and tolerance to salt and curing conditions. However, the efficacy of microbial nitrate reduction is influenced by several factors, including vegetable source, pH, incubation temperature, and salt concentration, which affect enzymatic activity and nitrite yield [4,40,45,61,62].

Recent studies have investigated the contributions of nitrate-reducing strains to in situ nitrite generation and color stabilization in naturally cured meat products. For example, *Staphylococcus hominis* WiKim0113 isolated from kimchi, reduced nitrate by 45% while simultaneously exhibiting antimicrobial effects against *Clostridium perfringens* and maintaining enzymatic activity across a wide pH and salt concentration range [63]. In a study by Szymański et al. [64], pork sausages cured with a reduced nitrite level (15 mg/kg) combined with *S. carnosus* ATCC 51365 (10^7^ CFU/g) exhibited improved redness and nitrosyl pigment content compared to sausages cured with 15 mg/kg nitrite alone and performed comparably to a 100 mg/kg nitrite control after 8 weeks of storage. Alternative species such as *Staphylococcus caprae* and *Pantoea agglomerans* have also demonstrated the ability to generate over 850 ppm of nitrite in fermentation models while maintaining salt and heat resistance, indicating broader microbial reservoirs for nitrate conversion in meat systems [61]. Lactic acid bacteria (LAB) such as *Lactobacillus plantarum* and *Lacticaseibacillus paracasei* have also been studied, exhibiting moderate nitrite production and improved visual redness when fermented with celery powder. However, they were typically less effective compared with traditional nitrate-reducing staphylococci [65,66]. In practical applications, mixed-strain cultures incorporating both staphylococci and LAB have been employed to achieve synergistic outcomes such as nitrate reduction, pH modulation, and microbial inhibition [45]. Table 2 summarizes alternative curing strategies for replacing traditional synthetic nitrite systems, including microbial reduction of nitrate-rich vegetables by starter cultures and the use of pre-converted vegetable-derived nitrite sources for clean-label meat products.

Recent studies demonstrated that nitrate-rich vegetable powders can serve as effective natural nitrite sources when combined with suitable starter cultures. Jeong et al. [37] examined ground pork sausages cured with Chinese cabbage powder (0.15–0.35%) combined with *S. carnosus*. Treatment with 0.25–0.35% Chinese cabbage powder exhibited curing efficiencies exceeding 80% with comparable redness values to those obtained with celery powder, while maintaining significantly lower residual nitrite levels than those observed after sodium nitrite treatment. Such findings indicate that Chinese cabbage powder may serve as a viable source of nitrate and nitrite when combined with microbial conversion systems. Guimarães et al. [72] examined cooked hams treated with Japanese radish in powder, juice, and pulp powder forms alongside *S. carnosus*-based starter cultures. The residual nitrite levels ranged from 9.8 to 11.0 mg/kg, with radish juice achieving hue angles comparable to those of a 150 ppm sodium nitrite control. Meanwhile, sensory evaluations indicated that appearance and aroma were similar to hams cured with 40 ppm sodium nitrite, suggesting that Japanese radish derivatives may facilitate acceptable curing performance in clean-label meat products.

Several studies have highlighted the adaptability of microbial nitrate-reduction systems, reporting successful pigment formation in meat products utilizing radish, celery, Swiss chard, or spinach in conjunction with *S. carnosus* or mixed starter cultures; however, variability in pigment intensity and oxidative stability has also been noted [34,36,38,70]. For example, Öztürk-Kerimoğlu and Serdaroğlu [70] observed that chard powder containing 150 ppm nitrate increased the residual nitrite levels but resulted in reduced redness compared with a synthetic nitrate control, suggesting incomplete pigment stabilization despite adequate nitrite production. Although these studies demonstrated the feasibility of microbial nitrate reduction systems, challenges persist concerning pigment consistency and oxidative stability. Notably, color development and oxidative stability depend on the type of vegetable, its physical form (e.g., juice vs. powder), and fermentation conditions. For instance, Ozaki et al. [38] reported that nitrite-free fermented sausages containing 1% radish powder exhibited reduced redness and elevated lipid oxidation, with thiobarbituric acid reactive substances (TBARS) levels reaching 2.76 mg malondialdehyde (MDA)/kg at 20 °C in the absence of antioxidants. These findings highlight the importance of co-applying antioxidant compounds and selecting suitable nitrate carriers to improve pigment development and oxidative stability in vegetable-based curing systems.

Overall, nitrate reduction using starter cultures is a promising strategy for clean-label meat curing, particularly when combined with well-characterized nitrate sources and carefully optimized processing parameters. Further advancements will require standardizing the nitrate content, enhancing the compatibility with natural antioxidants, and ensuring consistent product performance across diverse product categories.

#### 2.1.3. Pre-Converted Vegetable Powders and Brines

In contrast to microbial nitrate conversion, pre-converted vegetable nitrite systems provide a direct and standardized source of natural nitrite without relying on in situ microbial activity. As discussed in Section 2.1.1, nitrate-rich vegetables such as celery, radish, and spinach serve as the base materials for these systems. Among them, celery is the most frequently used owing to its consistently high nitrate content and fermentation stability, yielding nitrite concentrations of 15,000–20,000 mg/kg under controlled fermentation conditions [3,26]. Pre-converted systems are typically applied as dried powders or liquid brines and are particularly suitable for heat-treated or emulsified meat products, where microbial viability is limited. Furthermore, they enable more predictable nitrite delivery based on product design parameters [3,26].

Several studies have evaluated the curing efficacy of pre-converted vegetable powders. Rasmussen [74] investigated a model system combining pre-converted celery and acerola cherry powders, reporting the highest cured pigment concentration (22.57 ppm), reducing activity, and sulfhydryl concentration (3.50 mM) among the tested groups. However, the residual nitrite levels were higher than those in the sodium nitrite controls, suggesting possible limitations in nitric oxide regeneration or nitrite utilization. Posthuma et al. [43] extended this approach to emulsified beef sausages, reporting decreased residual nitrite levels while maintaining effective color development, indicating a synergistic interaction between the pre-converted nitrite and natural reductants. Patton [42] evaluated beef jerky treated with pre-converted celery and cherry powders; the use of cherry powder alone produced the highest redness value (a* ≈ 41), surpassing both the combined treatment and sodium nitrite control. These findings suggest that the enhanced cured color may also be attributed to synergistic antioxidant effects or pigment stabilization mechanisms, independent of nitrite levels.

In a study on fermented vegetable brines, Hwang et al. [35] revealed that extracts from lettuce, celery, and red beets yielded only modest cured color development, characterized by significant variability in the hue angles and limited nitrite retention. Conversely, fermented spinach extracts showed improved pigment formation and oxidative stability. In a comparable approach, fermented red beet and Swiss chard brines enhanced the cured color of cooked meat products, with the redness values approaching those of the synthetic nitrite controls [44,75]. However, the residual nitrite levels and markers of protein or microbial stability varied depending on the vegetable source, concentration, and co-applied antioxidants, highlighting the importance of optimizing vegetable selection, fermentation conditions, and antioxidant compatibility.

As outlined in Table 2, pre-converted vegetable systems have demonstrated promising efficiency in various clean-label meat products by directly providing natural nitrite. Their functional efficacy can be further enhanced through the incorporation of natural antioxidants, such as cherry or citrus extracts. However, key limitations remain, including inter-batch variability, unpredictable pigment interactions, and microbial stability concerns, especially in liquid-based pre-converted systems. To ensure consistent and reliable performance, future research should focus on standardizing the active nitrite content, optimizing antioxidant pairing strategies, and enhancing microbial safety measures.

### 2.2. Curing Accelerator Replacement Strategies

In traditional meat curing systems, curing accelerators are defined as compounds that promote the formation of nitrosyl hemochrome, the pigment responsible for the characteristic pink color of cured meat products, primarily by facilitating the reduction of nitrite to nitric oxide [76]. These accelerators are generally classified into two mechanistic categories: acidulants and reductants. Acidulants, including lactic, citric, and acetic acids, enhance nitric oxide generation by lowering the pH, thereby accelerating the curing reaction and improving microbial stability [76,77]. Conversely, reductants such as ascorbate and erythorbate function as electron donors, directly reducing nitrite while simultaneously providing antioxidative protection [3,4,54,76,78]. This dual functionality has rendered synthetic reductants indispensable in traditional meat products. However, the increasing consumer demand for clean-label alternatives has prompted active research into naturally derived compounds capable of mimicking the nitrite-reducing and antioxidative roles of synthetic curing accelerators [3,4]. Although synthetic ascorbate is generally considered safe, its chemically derived nature and the challenges associated with its labeling have spurred interest in the discovery of natural alternatives. Such alternatives primarily include vitamin C-rich reductants and polyphenol-based antioxidants, which can fulfill the functional roles typically provided by synthetic ascorbate [2,11]. Consequently, clean-label curing systems must strategically reproduce both the acidification and reduction pathways to maintain product quality without relying on conventional synthetic additives.

Among the available approaches, natural reductants rich in vitamin C, such as acerola, citrus peel, and cherry powder, have shown promise by providing natural ascorbic acid, which facilitates nitric oxide production and inhibits lipid oxidation. However, their effectiveness may vary depending on the composition and concentration of the ingredients [11,18,79]. Concurrently, polyphenol-based antioxidants derived from teas, herbs, and fruits, such as tea polyphenols, blackcurrant leaf extract, rosemary, and sage essential oil, have been shown to enhance cured pigment stabilization, provide oxidative protection, and reduce the formation of N-nitrosamines [14,29,30].

However, these compounds require meticulous adjustment of their composition and application levels because their sensory impact is dose dependent and may negatively affect flavor perception if not appropriately balanced. To enhance the functional reliability and address the limitations of individual ingredients, integrated approaches have been developed. These approaches combine nitrate-rich vegetable powders, nitrate-reducing starter cultures, and plant-derived reductants or antioxidants, aiming to emulate the synergistic interactions characteristic of conventional curing systems involving nitrite and ascorbate. Each approach presents distinct advantages and limitations contingent on the product type, ingredient compatibility, and processing conditions. Table 3 provides a functional classification of plant-based reductants and antioxidants, detailing their primary functionalities and observed effects on clean-label meat products.

#### 2.2.1. Vitamin C-Rich Sources as Natural Curing Accelerators

Synthetic curing accelerators such as sodium ascorbate and erythorbate have long been employed in meat processing owing to their dual functionality, facilitating the reduction of nitrite to nitric oxide, which binds to heme pigments to form nitrosyl hemochrome, and providing antioxidative protection by scavenging reactive oxygen species [3,76,78]. In the context of clean-label curing strategies, naturally derived ingredients rich in ascorbic acid have attracted considerable attention as potential alternatives for achieving these key functions.

Acerola is one of the most frequently studied natural sources of vitamin C for meat curing. Gubała and Migdał [11] reported that the inclusion of 0.025% acerola powder in cured pork sausages achieved an a* value of 16.50, which was nearly equivalent to that of sodium isoascorbate (a* = 16.56), while maintaining microbial stability during refrigerated storage. However, at a higher concentration (0.05%), reductions in cured color and increased microbial counts were observed, possibly due to pH-related effects or over-acidification. Cherry powder has also been investigated as a natural curing accelerator. Terns et al. [79] reported that when used alongside nitrate-rich celery powder and *S. carnosus*, cherry powder enhanced cured pigment development and a* values to levels comparable to those obtained with 156 ppm sodium nitrite. Posthuma et al. [43] further validated these findings using a pre-converted fermented vegetable system containing cherry powder, which improved nitrosyl hemochrome formation and color characteristics without synthetic additives. Citrus-derived powders, such as citrus peel extract powder (CEP), have shown favorable antioxidant properties in cured meat. According to Bae et al. [18], ground pork sausages containing 0.2% CEP and 0.4% Chinese cabbage powder had TBARS values comparable to those of sausages treated with 0.05% sodium ascorbate. However, the residual nitrite concentration in the CEP treatment remained high, indicating limited nitrite-reducing capacity despite acceptable redness.

Collectively, these studies suggest that acerola, cherry, and citrus powders are promising candidates as clean-label curing accelerators. Their efficacy depends heavily on appropriate inclusion levels, pH control, the presence of nitrate-rich vegetables, and the use of starter cultures. When these factors are optimized, vitamin C-rich sources can contribute to improved pigment stability, oxidative resistance, and reduced residual nitrite levels in meat products.

#### 2.2.2. Polyphenol-Based Antioxidants for Nitrite Reduction and Color Stability

In contrast to ascorbate-rich reductants that directly convert nitrite to nitric oxide, polyphenol-based antioxidants exert their effects through indirect mechanisms, primarily involving radical scavenging, metal chelation, and the inhibition of oxidative degradation pathways. These mechanisms help stabilize cured color, reduce lipid oxidation, and potentially mitigate N-nitrosamine formation, making them relevant for clean-label meat systems with reduced or absent nitrite levels [14,29].

Among polyphenol sources, tea-derived catechins and theaflavins have shown notable promise. Gao et al. [29] reported that the inclusion of tea polyphenols (300 mg/kg) in pork sausages containing 150 mg/kg sodium nitrite significantly increased redness (a* values) and nitrosyl hemochrome formation while reducing metmyoglobin and residual nitrite content. Notably, this treatment suppressed N-nitrosamine formation, supporting the potential use of tea extracts as functional antioxidants in nitrite-containing systems. Berry-derived phenolics, such as blackcurrant leaf extracts, have also demonstrated multifunctional effects. Wójciak et al. [89] showed that 150 mg/kg blackcurrant leaf extract maintained the nitrosohemochrome content while reducing TBARS formation in canned pork over six months of storage. The absence of detectable N-nitrosamines in the treated samples further highlights the long-term antioxidative and anti-nitrosative potential of the polyphenol systems. Herbal extracts, particularly rosemary, have been tested in various cooked or cured meat products [30,82,85]. Hoelscher et al. [30] evaluated a mixture of rosemary extract (0.10%), acerola powder (0.25%), and mixed tocopherols (0.03%) in pork sausages and revealed that the rosemary-containing treatments exhibited the lowest TBARS values among all treatments. However, no significant improvement in redness (a*) was observed, suggesting that rosemary functions more effectively as an antioxidant than as a nitrite-reducing agent.

In summary, polyphenol-based antioxidants derived from tea, berries, and herbs offer oxidative stability and partial pigment protection in nitrite-reduced systems. However, their functionality is generally indirect and depends on ingredient concentration, system compatibility, and processing conditions. While polyphenol-based antioxidants are not replacements for synthetic reductants, they play a supportive role in enhancing the stability and safety of clean-label cured meat products.

#### 2.2.3. Combined Systems of Natural Reductants and Antioxidants

Synergistic systems combining natural reductants and antioxidants have attracted increasing attention in response to the functional limitations of individual plant-derived curing agents. These integrated strategies are designed to serve as substitutes for the combined functional roles of nitrite and ascorbate in conventional curing systems, facilitating nitrosyl hemochrome formation and inhibiting lipid and pigment oxidation. Typical combinations include nitrate-rich vegetable powders, nitrate-reducing starter cultures (e.g., *S. carnosus*), and functional plant extracts that provide either ascorbic acid or polyphenolic antioxidants.

A representative example was reported by Choi et al. [28], who combined 0.2% white kimchi powder, 0.2% acerola juice powder, and 0.02% *S. carnosus* in naturally cured pork sausages. This combination achieved the highest curing efficiency (79.6%) and the lowest residual nitrite level (0.92 ppm) while maintaining an elevated nitrosyl hemochrome content (41.83 ppm) and suppressing TBARS values, indicating dual benefits in curing efficiency and oxidative stability. Serdaroğlu et al. [73] extended this approach to fermented sausages using pre-converted arugula extract (150 ppm nitrite equivalent) and barberry extract (200 ppm gallic acid equivalent). The integrated system significantly outperformed the sodium nitrite control (83.5% vs. 67.1% curing efficiency) and reduced TBARS and protein carbonyl levels, thereby confirming its multifaceted efficacy. Martínez et al. [68] introduced additional complexity to product design by developing a dry-cured chorizo using a mixture of nitrate-rich leafy vegetables (e.g., celery, spinach, and chard) and polyphenol-rich plant extracts, including acerola, citrus, and rosemary. This strategy reduced hexanal and nonanal concentrations while inhibiting *C. perfringens*, suggesting concurrent chemical and microbiological benefits. Yoon et al. [17] demonstrated that natural curing systems comprising 0.3% white kimchi powder, 0.05–0.10% rosemary or green tea extract powder, and *S. carnosus* could match the redness (a*) and nitrosyl hemochrome content of the synthetic nitrite control. Although the initial antioxidant protection was weaker, increasing the concentration of the polyphenolic extracts mitigated oxidative deterioration, indicating that optimizing extract ratios is critical for a consistent performance.

Taken together, combination systems that integrate natural nitrate donors, microbial reductants, and multifunctional antioxidants offer a promising clean-label strategy, although their success depends on optimizing ratios and minimizing sensory and process-related limitations.

### 2.3. Phosphate Replacement Strategies

Phosphates play a crucial role in the technological processing of meat products by improving their WHC, protein solubility, pH buffering, emulsification, and textural stability. These functionalities enhance cooking yield, juiciness, and structural integrity, thereby establishing phosphates as essential for maintaining product yield and processing consistency [93,94]. Nonetheless, increasing health concerns related to phosphate overconsumption, particularly among individuals with renal or cardiovascular disorders, have led to heightened consumer scrutiny and a growing demand for phosphate-reduced or phosphate-free alternatives [7,16,95]. In contrast to the substitution of nitrite or ascorbate, which is frequently addressed using specific plant-based or microbial alternatives, the replacement of phosphates presents distinct challenges. This is due to the lack of natural analogs that can match the ionic strength, chelation, and buffering properties of synthetic phosphates [5,16]. Consequently, recent strategies have shifted from chemical mimicry toward identifying functional analogues that approximate the role of phosphates in moisture retention, protein interactions, and texture development.

Natural phosphate substitutes have been broadly classified into six functional categories based on their origin and primary mechanisms of action: (1) Hydrocolloid-based binders, such as chia seed mucilage and sesame seed derivatives, enhance viscosity and moisture retention by forming gel-like networks within the meat matrix [96,97]. (2) Protein-based ingredients, including soy protein isolate, egg white, and blood plasma, facilitate gel formation and emulsification, thereby supporting structural integrity [98,99,100]. (3) Naturally derived calcium powders from eggshells and oyster shells elevate the pH and improve the WHC through alkaline buffering effects [12,101,102]. (4) Dietary fibers sourced from bamboo, soybean husks, and seaweed exhibit water-binding and swelling properties that enhance cooking yield and textural retention [103,104,105]. (5) Mushroom-derived replacers, particularly those from oyster and winter mushrooms, provide multifunctional benefits by enhancing emulsion stability, antioxidative protection, and moisture retention [106,107]. (6) Combination systems integrate two or more of these functional components, such as calcium powders combined with proteins or hydrocolloids, to achieve synergistic effects that better mimic the multifaceted functionality of synthetic phosphates [12,32,108].

Despite these advancements, complete phosphate replacement remains technically challenging. Most natural substitutes exhibit limitations in their buffering capacity and protein solubilization, often requiring supplementary processing strategies, such as ultrafine milling, optimized blending, or the application of non-thermal processing technologies [32,109,110,111]. Additional challenges include ingredient variability, cost implications, labeling constraints, and preserving desirable sensory characteristics in finished products. Section 2.3.1, Section 2.3.2, Section 2.3.3, Section 2.3.4, Section 2.3.5 and Section 2.3.6 systematically review the functional characteristics and representative applications of each category of natural phosphate substitutes. Furthermore, a summary of the comparative evaluations is presented in Table 4.

#### 2.3.1. Hydrocolloid-Based Replacers

Hydrocolloid-based ingredients have gained recognition as viable alternatives to phosphates in clean-label meat products, owing to their ability to enhance water retention, increase viscosity, and maintain emulsion stability. These natural polysaccharide systems, including chia seed mucilage, flaxseed gum, konjac gel, and carrageenan, primarily function by forming viscous networks that physically entrap moisture and fat, thereby improving the juiciness, yield, and texture of processed meat products [96,97].

However, the clean-label status of hydrocolloid-based ingredients is not consistent across global markets. For instance, carrageenan (E407) and konjac (E425) are designated food additives under EU legislation, requiring explicit declaration on product labels [118]. This may limit their use in clean-label product development depending on regional regulations and consumer expectations.

Although hydrocolloids do not fully substitute for the ionic strength or buffering capacity of synthetic phosphates, their physicochemical interactions with muscle proteins can partially restore their textural and water-holding properties under phosphate-free or phosphate-reduced conditions. Among the various hydrocolloid sources, chia seed mucilage has shown significant potential. Câmara et al. [96] demonstrated that the incorporation of 2% chia mucilage gel into low-fat Bologna-type sausages enhanced emulsion viscosity, reduced cooking loss, and yielded favorable sensory outcomes, including overall acceptability and a succulent mouthfeel, comparable to phosphate-treated controls. The robust gel-forming properties and positive consumer perception of chia further underscore its suitability for clean-label meat products. Other plant-derived hydrocolloids, such as carrageenan, pectin, and flaxseed gel, have also been examined for improving moisture retention, emulsification, and textural stability in meat products [116,119,120]. Nevertheless, their application requires careful dosage optimization, because excessive use may result in overgelling, increased hardness, or undesirable textural inconsistencies. Additionally, potential interactions with curing agents or natural antioxidants can affect color development and flavor perception, underscoring the importance of ingredient compatibility assessments during product development.

#### 2.3.2. Protein-Based Replacers

Proteins derived from both animal and plant sources have been investigated as viable functional alternatives to synthetic phosphates in clean-label meat products. Their ability to enhance water retention, stabilize emulsions, and form textures render them particularly valuable for products with reduced or absent phosphates. The efficacy of these proteins in restoring essential functionalities, such as minimizing cooking loss, supporting gel formation, and maintaining emulsion integrity, varies depending on their origin, structural properties, and degree of hydrolysis [99,100].

In comparative analyses of phosphate-free emulsified sausages, both blood plasma and soy proteins demonstrated cooking loss and emulsion stability comparable to those of phosphate-containing controls, whereas egg white and pea proteins exhibited moderate effects [99]. Conversely, gelatin and potato protein were significantly less effective, highlighting the importance of selecting appropriate protein sources based on the specific physicochemical requirements of the target product. The functional performance of hydrolyzed proteins has also been extensively investigated. Nuñez et al. [98] reported that 3% bovine skin gelatin hydrolysate with a 26.3% degree of hydrolysis significantly reduced cooking loss in phosphate-free chicken meat, achieving results comparable to those of 3% sodium tripolyphosphate (STPP)-treated controls. Notably, even hydrolysates with lower degrees of hydrolysis maintained a substantial water-binding capacity, suggesting that their efficacy is not solely attributable to buffering action but also to structural water entrapment. Plant-derived protein isolates, notably chickpea protein isolate (CPI), have demonstrated increasing potential as phosphate substitutes for clean-label meat products. Wang et al. [100] observed that the incorporation of 1.5–2.0% CPI enhanced water immobilization and emulsion stability in emulsified sausages through the formation of protein–protein and protein–water networks, achieving a functionality comparable to traditional phosphate systems. Such findings indicate that CPI may function as an effective clean-label binder by improving the WHC, emulsion stability, and textural properties such as hardness and cohesiveness. Recent advances in the use of modified myofibrillar proteins have further contributed to the development of phosphate replacement strategies. Kim et al. [112] demonstrated that the integration of frozen, pre-salted hot-boned pork with 2% myofibrillar protein improved the thermal stability, protein solubility, and cooking yield in phosphate-free emulsion-type sausages, achieving physicochemical properties comparable to those of phosphate-treated controls. In a subsequent study, Kim et al. [113] reported that grafting palatinose to myofibrillar proteins via the Maillard reaction further enhanced the pH stability, tenderness, and lipid oxidative stability in brined pork loin, underscoring its potential as a clean-label phosphate substitute.

Protein-based replacements can partially or, in some cases, effectively substitute for key functional roles of phosphates in meat processing. Animal-derived proteins typically demonstrate superior gelation and emulsifying properties, whereas plant-based proteins offer clean-label and nutritional advantages, albeit potentially necessitating formulation optimization to achieve comparable results. Future research should focus on improving their sensory acceptance and validating their performance under commercial manufacturing conditions.

#### 2.3.3. Natural Calcium-Based Replacers

Natural calcium-based powders, particularly those derived from eggshells and oyster shells, have attracted attention as potential clean-label alternatives to phosphates in processed meat products. Their primary mechanism involves increasing the pH of the meat system, thereby enhancing protein solubility, WHC, and gel strength, which are key functionalities of synthetic phosphates [101,102,109].

Among the examined sources, eggshell calcium (ESC) and oyster shell calcium (OSC) demonstrated superior performance in phosphate-reduced systems. Bae et al. [101] assessed four calcium sources, including eggshell calcium (ESC), oyster shell calcium (OSC), marine algae calcium, and milk-derived calcium, as full substitutes for 0.3% STPP in ground pork. ESC at 0.5% increased pH, reduced cooking loss, and improved redness, whereas both the ESC and OSC treatments achieved a texture quality comparable to that of the phosphate controls. Similarly, Cho et al. [109] revealed that a blend of 0.2% OSC and 0.3% ESC enhanced WHC and texture to levels similar to those of phosphate-treated products. In pork products cured with either synthetic nitrite or radish powder, Yoon et al. [13] showed that 0.5% OSC afforded a high cooking yield (96.8%) and minimized lipid and water separation. The high alkalinity of OSC (pH~9.9) likely promotes protein–water interactions and swelling. Compared to citrus fiber or dried plum powder, shell-derived calcium ingredients consistently provided a superior water-binding functionality. Furthermore, the effectiveness of calcium-based replacers was dose dependent. Bae and Jeong [102] reported that 0.3% OSC lowered cooking loss and patty shrinkage without negatively affecting texture, whereas 0.6% OSC led to excessive pH elevation and undesirable pigment changes, emphasizing the importance of dose optimization.

Despite their promising results, natural calcium powders have limitations including poor solubility, potential over-alkalization, and variability in mineral content and particle size. Strategies, including micronization, encapsulation, or a combination of emulsifiers and hydrocolloids, have been proposed to improve functionality and consistency. In summary, OSC and ESC are viable clean-label phosphate substitutes capable of partially restoring moisture retention and texture. Their successful application depends on precise dose control and their integration with complementary clean-label binding agents.

#### 2.3.4. Dietary Fiber-Based Replacers

Dietary fibers have garnered significant attention as potential clean-label alternatives to phosphates in processed meat products, primarily owing to their ability to enhance WHC, emulsion stability, and textural properties. Such functionalities arise from the structural complexity of polysaccharides, which interact with water, proteins, and fats to form cohesive gel networks. Such mechanisms are particularly advantageous for low-fat and phosphate-free meat products [93,103].

Among the various dietary fiber sources assessed for phosphate replacement, soybean husk dietary fibers (SHDF) and bamboo fibers have exhibited promising multifunctional properties. Araujo-Chapa et al. [104] reported that the incorporation of 1.5% SHDF into frankfurters enhanced water retention and textural properties while simultaneously contributing to calcium enrichment and sodium reduction. Notably, these functional benefits were achieved without compromising sensory acceptance, underscoring the dual role of SHDF in technological enhancement and nutritional fortification. Magalhães et al. [103] demonstrated that the addition of 5% bamboo fiber significantly reduced fat and water exudation in phosphate-free Bologna sausages. Furthermore, bamboo fiber treatment improved hardness and chewiness, with scanning electron microscopy revealing a denser and more compact protein gel network structure, similar to that observed in phosphate-containing products. Such findings highlight the potential of plant-derived fibers as an effective clean-label strategy for enhancing WHC, texture, and overall product stability in phosphate-free meat systems. Furthermore, the integration of flaxseed cake and seaweed-derived polysaccharides yielded promising results for emulsion-type meat products. Pinton et al. [116] demonstrated that replacing alkaline phosphate with 3% flaxseed cake preserved emulsion stability and WHC in mortadella, achieving sensory acceptance comparable to that of phosphate-treated controls. Similarly, Yuan et al. [105] reported that the incorporation of 1.0% seaweed dietary fiber significantly enhanced cooking yield and textural properties in frankfurters, which was attributed to improved protein gelation supported by hydrogen bonding and hydrophobic interactions. However, not all dietary fiber sources exhibit equal efficacy. Such variability may arise from differences in fiber type (e.g., soluble vs. insoluble), plant origin, and pH, which influence their water-binding and protein-interaction properties in meat systems [121,122]. Powell et al. [114,115] noted that commercial citrus fiber had limited effects on cooking yield and texture at inclusion levels of 0.25–1.0%, and in some cases, it adversely affected cohesiveness and resilience. Conversely, Weigel et al. [15] tested six freeze-dried vegetables in cooked sausages and revealed that Brussels sprouts, Red Kuri squash, and sweet corn (1.0–4.0%) significantly improved the WHC. Among these, Brussels sprouts exhibited textural properties comparable to those of the phosphate-treated controls, while sweet corn achieved similar sensory acceptance; such findings underscore their potential as clean-label phosphate alternatives.

In conclusion, dietary fibers possessing notable hydration, gelling, or emulsifying properties may partially substitute phosphates for specific functional roles in meat products. Nonetheless, their efficacy is highly dependent on the source material, concentration, and interactions with other components. Future research should prioritize the standardization of functional properties and investigate synergistic combinations with proteins or natural calcium-based binders to enhance reproducibility, optimize sensory quality, and facilitate their broader industrial application in clean-label meat products.

#### 2.3.5. Mushroom-Based Replacers

Edible mushrooms have been identified as multifunctional clean-label ingredients with the capacity to partially substitute synthetic phosphates in processed meat products. Their diverse composition, comprising dietary fibers such as chitin and β-glucans, essential amino acids, antioxidant polyphenols, and bioactive polysaccharides, contributes to the enhancement of WHC, protein stabilization, and oxidative protection [123,124,125]. Furthermore, mushrooms provide umami flavor and texture-enhancing properties, potentially minimizing the need for added flavorings in phosphate-free meat products.

Among various mushroom species, the winter mushroom has been extensively studied for its potential to replace phosphates in meat products. Choe et al. [107] incorporated 0.5–2.0% winter mushroom powder into emulsified sausages and observed increased pH values (6.13–6.33), reduced exudation of jelly and fat, and improved oxidative stability. Notably, at 2.0% inclusion, the exudate levels were lower than those in both the phosphate-free and phosphate-treated controls, while the TBARS values were significantly reduced at concentrations of 1.0% and above. Although slight softening of the texture was noted, sensory acceptability remained favorable up to a 1.5% inclusion level. Building on these findings, Jeong et al. [117] compared freeze-dried (FDP) and oven-dried (ODP) winter mushroom powders in beef patties and demonstrated that 1% FDP reduced cooking loss compared with phosphate-free controls, while ODP provided superior lipid oxidative stability. Although neither treatment fully restored textural strength, the combined benefits of moisture retention and oxidative protection underscore the potential of winter mushroom powder as a dual-function phosphate replacer. To enhance the versatility of mushroom-derived ingredients, Jo et al. [110] investigated the application of plasma-treated winter mushroom powder (PWMP) to canned ground ham. The incorporation of PWMP improved redness (a* values), increased nitrosyl pigment formation, and enhanced water retention compared to phosphate-free controls. These findings suggest that plasma-assisted modification may enhance the curing efficacy and stability-related properties of mushroom powder. Besides winter mushrooms, other edible species such as the oyster mushroom have also exhibited functional potential. Jung et al. [106] demonstrated that incorporating 2% oyster mushroom powder enhanced WHC, minimized cooking loss, and inhibited lipid oxidation in emulsified sausages. Microscopy and Fourier transform infrared spectroscopy analyses revealed that protein–polysaccharide interactions facilitated the formation of filamentous networks, thereby stabilizing emulsions and underscoring the potential of oyster mushroom powder as a clean-label phosphate substitute.

Collectively, mushroom-derived ingredients provide antioxidant functions, high WHC, and improved texture, making them viable candidates for partial phosphate replacement. Their multifunctionality can be further enhanced by combining with clean-label binders. Future research should focus on standardizing processing methods and improving reproducibility for broader industrial use.

#### 2.3.6. Combination Systems of Natural Phosphate Replacers

Combination systems represent a strategic approach for substituting synthetic phosphates by incorporating multiple clean-label ingredients with complementary functionalities. These systems typically integrate natural calcium powders, proteins, dietary fibers, and hydrocolloids to fulfill the extensive range of technological functions provided by phosphates, including WHC, pH modulation, emulsion stability, and textural enhancement [12,32].

Cho and Jeong [12] evaluated a phosphate alternative blend comprising 0.20% oyster shell calcium, 0.30% eggshell calcium, and 0.25% protein-based binders (whey protein concentrate (WPC), soy protein, or collagen) in pork products. Treatment with WPC or collagen significantly reduced cooking loss and enhanced textural properties, achieving results comparable to those of products treated with 0.3% STPP. Using a natural calcium–hydrocolloid combination, Kavuşan et al. [108] applied a combination of 0.50% eggshell calcium and 0.25% low-methoxyl pectin gel to restructured turkey steaks. This system improved protein solubility, WHC, and cooking yields that matched or exceeded those of the STPP-treated controls. Such enhancements were primarily attributed to the synergistic interaction between the pH-elevating effect of calcium and the gelling action of pectin, which contributed to the stabilization of the meat product structure. Pinton et al. [32] investigated a composite system comprising 2.5% bamboo fiber, 1.0% isolated pea protein, and 1.0% mushroom powder to produce low-sodium phosphate-free mortadella. This treatment enhanced emulsion stability and textural properties to levels comparable to those of the phosphate-containing controls. Although the inclusion of mushroom powder contributed to flavor enhancement, it also resulted in increased lipid oxidation and slight discoloration. Such a finding highlights the necessity for careful optimization of the sensory quality and oxidative stability of composite formulations.

Taken together, these findings indicate that functional synergy among natural ingredients can enhance the performance of clean-label meat products. By selecting and combining components with distinct yet complementary roles, such as pH buffering, emulsification, gel formation, and water retention, developers can tailor composite systems to meet specific product requirements. However, successful application requires careful optimization of ingredient ratios, the assessment of inter-component interactions, and control of processing variables to mitigate potential trade-offs such as oxidative instability or flavor imbalance.

In conclusion, combination systems are increasingly recognized as promising approaches for replacing the multifunctional roles traditionally provided by phosphates in meat products. Although no single natural ingredient can serve as a full replacement for the multifunctional roles of phosphate, well-designed integrated systems provide clean-label alternatives capable of preserving product quality, structural integrity, and consumer acceptance.

### 2.4. Non-Thermal Processing Technologies for Clean-Label Meat Products

Beyond additive substitution strategies, several non-thermal processing technologies have been explored to support the development of clean-label meat products. High-pressure processing (HPP), ultrasound (US), and cold plasma (CP) offer unique mechanisms that can improve microbial stability, physicochemical properties, and curing reactions. These approaches are particularly relevant in systems with reduced nitrite, ascorbate, or phosphate, where technological interventions may compensate for the partial loss of additive functionality. The subsections that follow summarize representative findings and highlight the synergistic potential of combining these methods with natural alternatives.

#### 2.4.1. High-Pressure Processing (HPP)

HPP is a non-thermal technology that has been shown to enhance the functional and microbiological stability of clean-label meat products. By applying pressures ranging from 100 to 600 MPa, HPP effectively inactivates spoilage and pathogenic microorganisms without causing the thermal degradation typically associated with conventional heat treatments. This process preserves the desirable sensory and nutritional qualities while ensuring microbial safety. HPP induces structural modifications in muscle proteins, thereby improving the WHC, emulsion stability, and textural properties, which are traditionally supported by synthetic phosphates, nitrite, and ascorbate [126,127,128,129].

Emerging evidence suggests that HPP may work synergistically with natural ingredient systems to enhance clean-label meat products. HPP facilitates protein gelation and improves oxidative stability, particularly in meat products enriched with natural antioxidants or those subjected to salt or nitrite reduction [126,127,130]. In phosphate-reduced meat emulsions, Lee et al. [131] demonstrated that the combination of 3% sea tangle powder with 100 MPa HPP significantly improved cooking yield and WHC, achieving results comparable to phosphate-treated controls. Similarly, Zheng et al. [132] revealed that pressure-assisted heating at 100–300 MPa enhanced WHC and facilitated the formation of porous gel networks in chicken batters. However, excessive pressure (400 MPa) compromised protein integrity and reduced the water-holding performance, highlighting the need for pressure optimization. In a hybrid phosphate replacement strategy, Thangavelu et al. [133] employed HPP at 150 MPa on Irish breakfast sausages treated with US-treated coffee silver (CSS). The integration of 0.2% STPP and 0.8% CSS resulted in an enhanced emulsion stability, textural quality, and color attributes, indicating that HPP can effectively facilitate composite or partial phosphate replacement strategies by improving moisture retention and protein functionality.

Besides phosphate replacement, HPP has also been investigated as an alternative strategy for nitrite reduction. Lee et al. [134] demonstrated that the application of HPP combined with 1–2% vinegar enhanced WHC, reduced lipid oxidation, and inhibited *C. perfringens* growth in nitrite-free emulsified sausages. Similarly, Pietrasik et al. [135] observed that a 600 MPa treatment for 3 min extended the microbial shelf life for up to 12 weeks in naturally cured restructured hams. However, reduced flavor acceptability was observed in celery powder-treated products, underscoring the importance of optimizing ingredient compatibility in clean-label curing systems.

Collectively, these studies indicate that HPP can enhance the physicochemical and microbial stabilities of natural curing systems. However, excessive pressure can cause oxidative damage or pigment degradation. Strategic incorporation of antioxidants or protective ingredients may mitigate these adverse effects and ensure product quality. As a scalable, commercially viable, and clean-label-friendly processing method, HPP has significant potential for the development of clean-label meat products. However, its optimal application requires careful formulation design and compatibility with natural replacers.

#### 2.4.2. Ultrasound (US) Processing

US processing is a non-thermal physical technology with considerable potential for enhancing the functionality of natural additives in clean-label meat products. When applied at low frequencies (typically 20–40 kHz) and high intensities, US induces acoustic cavitation, which is characterized by the formation, growth, and collapse of microbubbles within the medium. This phenomenon generates localized turbulence, intense shear forces, and transient thermal effects, collectively promoting solute dispersion, protein unfolding, and microstructural modifications in meat products [136,137].

The mechanistic advantages of US in meat processing are predominantly linked to its ability to extract and reorganize salt-soluble myofibrillar proteins. Such structural modifications enhance protein–water interactions, improve emulsification, and bolster system cohesion, thereby compensating for the absence of phosphate-based emulsifiers and enhancing WHC [130,136,137]. In curing systems, US has been documented to expedite diffusion and improve the uniform retention of solutes, such as nitrate, nitrite, and salt. This is particularly beneficial in whole-muscle products, where sponge effects and acoustic streaming enhance internal distribution and curing uniformity [136,137].

Despite its functional benefits, excessive or prolonged US exposure may accelerate oxidative degradation and induce protein denaturation, resulting in off-flavors, textural softening, and reduced oxidative stability. Therefore, careful optimization of the treatment parameters, including amplitude, duration, and temperature, is crucial for preserving product quality and maximizing functional efficacy [138,139]. Numerous studies have indicated that US treatment can enhance the functionality of meat products with reduced phosphate and nitrite contents. Cichoski et al. [140] demonstrated that US treatment (25 kHz, 60% amplitude, 5.5 min) significantly improved the emulsion stability, cohesiveness, and cooking yield of phosphate-free pork emulsions without inducing lipid oxidation. Similarly, Pinton et al. [141] revealed that US treatment (18 min) substantially enhanced the stability, cooking yield, and pH of phosphate-reduced meat emulsions. However, these advantages diminish with shorter exposure times and in phosphate-free meat systems, suggesting that the effectiveness of US treatment may be limited to a specific processing window. Building on these findings, Pinton et al. [142] investigated the combined effects of high-power US (25 kHz, 60% amplitude, 27 min) and bamboo fibers (2.5–5%) in phosphate-free meat emulsions. The addition of 2.5% bamboo fiber yielded the most favorable improvements in emulsion stability and cohesiveness, effectively compensating for the absence of phosphates. Nevertheless, US treatment increased lipid oxidation in all phosphate-free samples, highlighting the necessity for complementary antioxidant strategies when eliminating phosphates.

In conclusion, US is a promising approach for enhancing the physicochemical and structural integrity of reformulated meat products without synthetic phosphates or nitrites. However, its effectiveness is significantly influenced by the product composition and necessitates meticulous parameter optimization to prevent adverse quality effects. Consequently, US is optimally employed as part of a multicomponent clean-label strategy, particularly in combination with functional proteins, fibers, and antioxidants.

#### 2.4.3. Cold Plasma (CP) Technology

CP technology has emerged as a promising non-thermal intervention for clean-label meat curing, owing to its ability to generate reactive nitrogen species in situ under ambient or mild conditions without direct addition of synthetic nitrite. By producing reactive oxygen and nitrogen species, including nitric oxide, nitrogen dioxide, and ozone, CP initiates curing reactions and facilitates pigment formation [139,143]. In contrast to fermentation-based nitrate conversion, which relies on time-intensive microbial activity, CP enables rapid and controllable nitrite formation through interactions among the gas, liquid, and substrate components. This offers enhanced processing efficiency and control over cured color development, particularly in products targeting standardized nitrosyl hemochrome levels [129,139]. Moreover, CP can be applied either directly to meat products or indirectly by treating natural substrates, such as vegetables or protein powders, to create functional nitrite-rich ingredients, thereby broadening its application scope in clean-label curing systems [110,144,145].

Recent studies have highlighted the potential of the CP technology as a source of natural nitrites for meat curing applications. Jo et al. [110] demonstrated that atmospheric non-thermal plasma treatment of winter mushroom homogenate produced 4.87 g/kg of nitrite, facilitating its use at 1% concentration in ground hams. The treated powder achieved cured color development and oxidative stability comparable to those of conventional phosphate/nitrite treatments. However, the increased fat exudation and elevated hardness indicated the incomplete recovery of phosphate-like functional properties. In a subsequent study, Jo et al. [145] reported that CP-treated cauliflower powder achieved nitrite levels and cured color comparable to those of synthetic nitrite while significantly reducing N-nitrosopyrrolidine formation, highlighting its potential in mitigating nitrosamine risks in cured meat products. Besides pretreatment of ingredients, direct application of CP to meat products yielded promising results. Chen et al. [146] reported that atmospheric plasma treatment enhanced redness and significantly reduced the TBARS values in roasted lambs. Importantly, a 30 min plasma treatment without nitrite addition produced a volatile compound profile closely resembling that of conventionally cured samples and achieved comparable sensory acceptance. Beyond direct applications, the treatment of functional ingredients with CP has also been investigated. Marcinkowska-Lesiak et al. [144] applied non-thermal air plasma to egg white solutions, which were subsequently incorporated into pork liver pâté. The plasma-treated egg whites effectively facilitated the formation of cured pigments and enhanced oxidative stability without negatively affecting the pH or texture. Meng et al. [147] demonstrated that a CP-treated phosphate solution (CP-PBS) could partially replace nitrite in smoked sausages. The combination of 30–70% sodium nitrite with CP-PBS preserved redness and sensory attributes, comparable to those of sausages cured with 75 mg/kg sodium nitrite, while significantly reducing residual nitrite concentrations.

Although CP has considerable potential for nitrite-free curing, it is important to recognize certain limitations. CP alone does not fully achieve the broad functional performance provided by synthetic phosphates, particularly in terms of WHC and emulsion stability. Additionally, sensory outcomes can vary significantly depending on plasma parameters such as the gas composition, voltage, treatment duration, and medium. Therefore, meticulous optimization and ingredient compatibility are crucial to ensure consistent product quality. Furthermore, the establishment of harmonized standards for CP-treated ingredients would facilitate broader industrial applications.

In conclusion, CP technology represents a unique approach among non-thermal methods for clean-label meat production. When combined with complementary technologies, such as HPP, US, and CP can enhance the functional efficacy of natural ingredients by facilitating nitrite-free curing through in situ pigment generation. The strategic integration of these methods may yield synergistic advantages that support the partial substitution of synthetic additives while preserving product quality.

## 3. Challenges and Future Directions

Despite notable advancements in the development of clean-label curing systems, several scientific, technological, and regulatory challenges continue to hinder their widespread industrial adoption. These challenges arise from the inherent variability of natural ingredients, the difficulties in achieving the multifunctional performance of synthetic additives, and the lack of harmonized regulatory standards for clean-label claims.

### 3.1. Key Challenges in Clean-Label Meat Product Development

Despite significant advancements in research, the development of clean-label meat products continues to face substantial technological and practical challenges, with a primary impediment being the functional inconsistencies of natural additives in fulfilling the roles the roles of conventional curing systems. Such challenges stem from ingredient variability, sensory trade-offs, limited multifunctionality, process incompatibility, and regulatory ambiguity [11,16,139].

One key issue involves the unstable performance of natural nitrate and reductant systems, especially in achieving consistent nitrite conversion and a stable cured color under varying conditions. Bae et al. [18] reported that sausages treated with citrus peel extract exhibited higher residual nitrite levels than those treated with sodium ascorbate, indicating incomplete nitrite reduction or limited compatibility with the meat curing system. Similarly, natural nitrite sources, such as fermented beetroot and spinach, showed low nitrite retention and irregular color development, resulting in product variability [35,75]. Sensory limitations further complicate clean-label applications. While many natural extracts provide functional benefits, they can also introduce off-flavors or affect texture. Šojić et al. [14] reported bitterness in fermented sausages with high levels of sage oil, while Hoelscher et al. [30] observed overlapping aromas in pork sausages combining rosemary, acerola, and tocopherol. Similar sensory drawbacks have been reported in systems that incorporate fermented vegetables or plant powders [15,148]. Furthermore, several natural additives lack the multifunctionality of their synthetic counterparts. For example, phosphate replacers, such as seaweed or citrus fiber, enhance water retention but fail to deliver the buffering or gel-forming functions needed to maintain structural integrity [105,114,115]. Likewise, polyphenol-based antioxidants, such as pomegranate peel or pitaya extract, reduce lipid oxidation (TBARS) but do not contribute to cured pigment development, limiting their utility in complete nitrite replacement [86,149].

Beyond technical concerns, the regulatory status of natural additives represents a critical barrier to the broader adoption of clean-label strategies. Regulatory interpretations of “clean label” are not harmonized across countries or product categories. For example, recent legislative updates in the European Union [22] have reduced the maximum allowable nitrite levels in meat products. Moreover, if nitrate-rich vegetable extracts are added for preservation or curing purposes, they are classified as functional food additives and are subject to mandatory labeling, according to EU guidance [21]. These legal interpretations directly influence the clean-label designation of such ingredients and necessitate product-specific regulatory assessments and regionally adapted labeling strategies.

Finally, variation in plant-based ingredients due to differences in species, seasonal conditions, and processing methods makes it difficult to achieve batch-to-batch consistency and standardization [35,60]. Combined with high production costs and limited scalability, these factors restrict broader industrial applications [150].

### 3.2. Strategic Research and Development Priorities

To address these challenges, future research and industrial practices should prioritize ingredient standardization, robust sourcing protocols, and processing optimization to ensure consistent functionality and reproducibility in clean-label meat systems. One promising direction involves the application of multifunctional ingredient systems that combine pH buffering, emulsification, and antioxidation properties. Such systems, often composed of natural calcium salts, dietary fibers, and proteins, have shown potential in mimicking phosphate functions; however, their broader adoption requires further validation under industrial-scale conditions [12,108]. Although these blended systems show promise, further validation under industrial-scale processing conditions is required to ensure their practical viability. In parallel, ensuring the safety and shelf stability of clean-label meat products is another fundamental requirement. Systematic storage trials should be conducted to evaluate indicators such as residual nitrite, TBARS, pigment degradation, and sensory decline, particularly for systems using pre-converted vegetable nitrites or polyphenol-based extracts [15,75,86,149]. Additionally, terminological clarity is also necessary to avoid confusion and strengthen consumer trust. Labeling including “no nitrite added” or “natural curing” should be standardized and clearly communicated to align regulatory and market expectations [3,19,24]. The definition of “clean label” varies across scientific, regulatory, and consumer perspectives. While some regions emphasize additive labeling (e.g., presence of E-numbers), others focus on avoiding synthetic ingredients and favoring natural, familiar, or minimally processed components. This divergence reflects the absence of a universal regulatory standard and the influence of consumer perception, which often associates clean label with simple, recognizable ingredients [3,16,20]. A harmonized yet flexible framework is needed to align regulatory compliance with consumer expectations. Although natural curing systems and non-thermal processing technologies offer promising functional advantages, their widespread industrial adoption remains constrained by economic and scalability challenges. For example, natural nitrite sources such as cultured celery powder could cost 15 to 20 times more than their synthetic counterparts, thereby introducing significant formulation cost burdens in commercial production [1]. Similarly, capital investment for HPP equipment may range from USD 500,000 to over 3 million, depending on system capacity and specifications, restricting its implementation primarily to large-scale processors [1]. To support broader application, future studies should incorporate techno-economic assessments that evaluate ingredient costs, processing investments, and cost–performance trade-offs across various product categories. From a sustainability perspective, the valorization of agro-industrial byproducts, such as fruit peels, vegetable trimmings, and seed hulls, can offer the dual benefits of waste reduction and ingredient functionality. Their integration into clean-label meat systems through green technologies may enable environmentally responsible and cost-effective solutions [15,150].

In summary, advancing clean-label meat processing requires multidisciplinary efforts involving ingredient optimization, scalable processing validation, robust safety assurance, regulatory clarity, and sustainability integration. A coordinated strategy that balances technological performance, sensory quality, and consumer expectations is essential for the long-term success of clean-label meat products.

## 4. Conclusions

Growing demand for clean-label meat products has driven the replacement of synthetic nitrite, ascorbate, and phosphate with natural alternatives. This review summarized advances in plant-derived nitrates, natural reductants, and phosphate replacers, as well as non-thermal technologies that support their functional efficacy. Promising strategies include pre-converted nitrate systems, starter cultures, plant-based reductants, and phosphate replacers such as calcium, protein, or fiber-based ingredients. However, challenges remain in standardization, sensory optimization, and regulatory acceptance.

Notably, the absence of a universally accepted definition of “clean label” complicates both regulatory alignment and industrial implementation. To enable practical application in meat products, future research should aim to integrate ingredient functionality with technological innovation while aligning clean-label development with safety, consumer perception, and sustainability goals.

## Figures and Tables

**Table 1:** Nitrate-rich vegetables relevant to clean-label meat curing.

Vegetables	Nitrate Content (mg/kg)		Countries	References
	Mean	Range		
Celery (*Apium graveolens* L.)	1291	54–3770	China	[46]
	2610	1390–3370	Iran	[47]
	2422	101–6303	Republic of Korea	[48]
	1496	20–4269	USA	[49]
Chinese cabbage (*Brassica rapa* subsp. *pekinensis*)	1135	275–2177	China	[46]
	2117	1965–2195	Republic of Korea	[34]
Lettuce (*Lactuca sativa* L.)	1035	170–3184	Croatia	[50]
	1063	203–2767	Slovenia	[51]
	1387	33–3944	Republic of Korea	[48]
	851	79–2171	USA	[49]
Radish (*Raphanus sativus* L.)	475	203–904	China	[46]
	6260	3930–8980	Iran	[47]
	3477	3238–3981	Republic of Korea	[34]
	2400	1800–2800	Tunisia	[52]
Spinach (*Spinacia oleracea*)	1057	169–2351	Croatia	[50]
	2036	96–3559	Italy	[53]
	2124	18–6720	Republic of Korea	[48]
	2797	65–8000	USA	[49]
Swiss chard (*Beta vulgaris* L.)	1914	233–3558	Croatia	[50]
	1728	1026–2430	Italy	[53]

**Table 2:** Strategies using plant-based nitrate or nitrite sources for clean-label meat curing.

Replacement Strategies	Meat Products	Natural Ingredients and Dosages	Starter Cultures	Major Results	References
Nitrate conversion with a starter culture	Turkish fermented beef sausage (sucuk)	Beetroot powder (0.12%, 0.24%, and 0.35%)	*Staphylococcus carnosus*, *Pediococcus acidilactici*, *Lactobacillus sakei*	a* values increased to 18.6 (vs. 17.5 in 150 mg/kg sodium nitrite control) with 0.35% beetroot powder addition, while lactic acid bacteria counts increased, and TBARS levels were elevated at higher doses.	[67]
Nitrate conversion with a starter culture	Spanish chorizo	Lettuce extract 3000 ppm + arugula extract 1500 ppm + watercress extract 1500 ppm, spinach extract 3000 ppm + celery extract 3000 ppm, chard extract 3000 ppm + beet extract 3000 ppm	*Pediococcus* spp., *Staphylococcus xylosus*, *S. carnosus*	All natural extracts exhibited strong antioxidant and antimicrobial activities against *Clostridium perfrigens*, effectively replacing synthetic nitrate.	[68]
Nitrate conversion with a starter culture	Cured pork sausage	Radish powder (0.15% and 0.30%), celery powder (0.30%)	*S. carnosus*	A 0.30% radish powder treatment with 4 h incubation resulted in a residual nitrite content (39.62 ppm) similar to the 0.01% sodium nitrite control (41.56 ppm), along with enhanced curing efficiency.	[69]
Nitrate conversion with a starter culture	Ground pork sausage	Chinese cabbage powder, radish powder, spinach powder (0.4% each)	*S. carnosus*	Radish and Chinese cabbage powders maintained a curing efficiency above 80%, with a* values of 10.74 and 10.40, respectively; spinach yielded lower a* (8.17 vs. 10.72 in the 150 ppm nitrite control) despite high nitrosyl hemochrome.	[36]
Nitrate conversion with a starter culture	Pork sausage	White kimchi powder (0.2% and 0.4%), acerola juice powder (0.1% and 0.2%)	*S. carnosus*	The combination of white kimchi powder and acerola juice powder showed similar a* values (a* = 10.59–10.73 vs. 10.59 in 100 ppm nitrite-added control), enhanced cure efficiency and nitrosyl hemochrome formation, and lower residual nitrite levels.	[28]
Nitrate conversion with a starter culture	Ground pork sausage	Chinese cabbage powder (0.15%, 0.25%, and 0.35%), celery powder (0.40%)	*S. carnosus*	Chinese cabbage powder (0.25–0.35%) yielded a curing efficiency above 80%, with increased a* values (10.06–10.12 vs. 8.85 in 100 ppm nitrite control) and lower residual nitrite compared to the nitrite-added control.	[37]
Nitrate conversion with a starter culture	Heat-treated sucuk	Chard powder (75 ppm and 150 ppm as nitrate)	*P. acidilactici*, *Lactobacillus plantarum*, *S. carnosus*	The 150 ppm nitrate containing chard powder produced the highest residual nitrite but decreased a* values compared to synthetic nitrate control.	[70]
Nitrate conversion with a starter culture	Cooked restructured ham	Radish powder (1%), radish pulp powder (1%), radish juice (9%)	*S. carnosus* ssp. + *S. carnosus*	Radish pulp powder showed a similar hue with residual nitrite of 3.04–14.08 ppm; 120 min incubation enhanced nitrate reduction.	[71]
Nitrate conversion with a starter culture	Cooked restructured ham	Japanese radish powder (0.5%), Japanese radish juice (3.0%), Japanese radish pulp powder (0.5%)	*S. carnosus* ssp. + *S. carnosus*	Japanese radish juice (3.0%) resulted in hue angles similar to the 150 ppm nitrite control and appearance/aroma scores comparable to hams cured with 40 ppm sodium nitrite; residual nitrite ranged from 9.8 to 11.0 mg/kg	[72]
Nitrate conversion with a starter culture	Fermented cooked sausage	Radish powder (0.5% and 1.0%), oregano essential oil (0.01%)	*S. xylosus*, *Pediococcus pentosaceus*, *S. carnosus*	Radish powder with or without oregano essential oil yielded a* values of 11.1–12.5 at day 60. The 0.5% radish group showed higher redness than the nitrite control (a* = 11.5), and sensory quality was acceptable; oregano essential oil improved aroma.	[38]
Nitrate conversion with a starter culture	Pork sausage	White kimchi powder (0.30%), celery juice powder (0.30%), green tea and rosemary extract powder (0.05% and 0.10% each)	*S. carnosus*	White kimchi powder exhibited similar a* values (8.40–8.71 vs. 8.66 in nitrite-added control), as well as comparable nitrosyl hemochrome, total pigment, and curing efficiency; antioxidant activity improved at 0.10% extract level, but TBARS remained high.	[17]
Nitrate conversion with a starter culture	Emulsion-type sausage	Dongchimi powder (0.25%, 0.35%, 0.45%, and 0.55%)	*S. carnosus*, *Staphylococcus vitulinus*	Dongchimi powder (0.55%) exhibited similar residual nitrite, nitrosyl hemochrome, total pigment, and curing efficiency (≥84%) to 0.01% nitrite control.	[33]
Nitrate conversion with a starter culture	Ground pork sausage	Chinese cabbage filtered juice powder, Chinese cabbage crushed powder, radish filtered juice powder, radish crushed powder (0.4% each)	*S. carnosus* ssp. *+ S. carnosus*	Radish groups achieved a* values of 9.22–9.25, similar to the 0.01% nitrite control (a* = 9.34), with curing efficiency exceeding 75% and TBARS values below 0.15 mg malondialdehyde (MDA)/kg.	[34]
Nitrate conversion with a starter culture + pre-converted powder	Heat-treated fermented sausage	Pre-converted arugula extract (1.5%), arugula extract (1.2%), barberry extract (0.5%)	*S. carnosus*, *L. plantarum*, *P. acidilactici*	Pre-converted arugula and barberry powders exhibited the highest curing efficiency (83.5%), with barberry enhancing oxidative stability.	[73]
Nitrate conversion with a starter culture	Ground pork products	Radish powder (0.4%)	*S. carnosus* ssp. *+ S. carnosus*	Radish powder yielded an a* value of 9.86, comparable to the nitrite-added control (a* = 9.87), with similar nitrosyl hemochrome content and TBARS levels.	[13]
Pre-converted vegetable powder	Cured meat model system	Pre-converted celery juice powder (0.44%), cherry powder (0.40%)	None	Pre-converted celery juice powder and cherry powder increased cured meat pigment and curing efficiency and decreased residual nitrite compared to celery alone.	[43]
Pre-converted vegetable powder	Restructured beef jerky	Pre-converted celery powder (0.040%), cherry powder (0.004%)	None	Pre-converted celery and cherry powders developed cured color comparable to nitrite-treated products, reduced residual nitrite levels, and demonstrated high acceptability in both visual appearance and overall sensory attributes, including tenderness, juiciness, and flavor.	[42]
Pre-converted vegetable powder	Model curing system	Pre-converted celery juice powder (10–200 ppm nitrite concentration), acerola cherry powder (0.5%, 2.76 mM ascorbic acid)	None	Pre-converted celery and acerola cherry powders yielded the highest cured meat pigment (22.57 ppm), along with high reducing activity and sulfhydryl group levels; residual nitrite remained elevated.	[74]
Pre-converted vegetable brine	Pork emulsion sausage	Fermented red beet extract (5% and 10%)	*S. carnosus*	The 10% fermented red beet extract combined with ascorbic acid decreased the a* values (5.61 vs. 7.11 in 0.015% nitrite control) while achieving low TBARS (0.14 mg MDA/kg) and sensory quality comparable to nitrite-added control.	[75]
Pre-converted vegetable brine	Cooked pork sausage	Fermented spinach extract, fermented lettuce extract, fermented celery extract, fermented red beet extract (3% each)	*S. carnosus*	Fermented spinach extract resulted in the highest a* values (a* = 8.9), while fermented lettuce extract exhibited the lowest redness (a* = 3.5) compared to the nitrite-added control (a* = 10.1)	[35]
Pre-converted vegetable brine	Cured pork loin	Fermented Swiss chard solution (10%, 20%, 30%, and 40%)	*S. carnosus*	The application of 40% fermented Swiss chard solution significantly increased the a* value (9.08 vs. 8.47 in 120 ppm sodium nitrite control), along with the highest curing pigment content (40.46 ppm) and curing efficiency (90.2%), while maintaining sensory acceptability.	[44]

**Table 3:** Functional classification of plant-based reductants and antioxidants used in natural curing systems.

Replacement Strategies	Functional Activities	Meat Products	Plant-Based Sources and Addition Levels	Major Results	References
Vitamin C-rich plant sources	Curing accelerator	Emulsified cooked sausage	Cherry powder (0.2%)	Cured pigment concentration increased to 113.1 ppm on day 0, and color and sensory traits matched those of 156 ppm nitrite control.	[79]
Vitamin C-rich plant sources	Curing accelerator	Restructured beef jerky	Cherry powder (0.004%)	Residual nitrite levels and sensory attributes were comparable to nitrite/erythorbate-treated products.	[42]
Vitamin C-rich plant sources	Curing accelerator	Cured meat model system	Acerola cherry powder (0.5%, 2.76 mM ascorbic acid)	The treatment showed the highest cured pigment content (22.6 ppm), enhanced reducing activity, and increased sulfhydryl group levels (3.5 mM) at 200 ppm nitrite.	[74]
Vitamin C-rich plant sources	Curing accelerator	Emulsified beef sausage	Cherry powder (0.4%)	Cured meat pigment increased to 124.8 ppm (vs. 51.9 ppm in celery-only treatment), and percent cured pigment reached 72.3%.	[43]
Vitamin C-rich plant sources	Curing accelerator	Cured pork sausage	Dried acerola powder (0.025% and 0.050%)	A 0.025% acerola treatment maintained redness (a* = 16.50) similar to 0.01% sodium isoascorbate, with no adverse effects on sensory quality or texture.	[11]
Vitamin C-rich plant sources	Curing accelerator, antioxidant	Naturally cured pork sausage	Grapefruit, lemon, orange, mandarin peel extract powders (0.1% each)	Cured pigment formation, curing efficiency, and thiobarbituric acid reactive substances (TBARS) values were comparable to sodium ascorbate control.	[18]
Polyphenol-based antioxidants	Antioxidant	Dry-cured bacon	Green tea polyphenols (0.03%), grape seed extract (0.03%), green tea polyphenols (0.015%) + grape seed extract (0.015%)	Green tea polyphenols reduced TBARS (0.21 vs. 0.44 mg malondialdehyde (MDA)/kg), residual nitrite, biogenic amines, and N-nitrosodimethylamine while achieving the highest sensory scores.	[80]
Polyphenol-based antioxidants	Antioxidant	Cooked cured sausage (*Botifarra catalana*)	Mixed tocopherol extract (0.02%)	No significant effect was observed on TBARS or acceptability, although red color was preserved during storage.	[81]
Polyphenol-based antioxidants	Antioxidant	Pork sausage	Lyophilized rosemary extract, rosemary essential oil (0.2% each)	Lyophilized rosemary extract reduced TBARS by up to 47.3% after 49 days, outperforming rosemary essential oil and synthetic antioxidants.	[82]
Polyphenol-based antioxidants	Antioxidant	Chinese-style sausage	Clove extract (0.25%, 0.50%, 1%, and 2%)	Clove extract inhibited TBARS and protein carbonyl formation during storage of 21 days while preserving redness and textural properties.	[83]
Polyphenol-based antioxidants	Antioxidant	Frankfurter-type sausage	Green tea extract, *Urtica dioica* L. extract, olive leaf extract (500 ppm each)	Green tea extract exhibited the strongest TBARS inhibition, while *Urtica dioica* L. extract showed the highest sensory acceptability and microbial stability.	[84]
Polyphenol-based antioxidants	Antioxidant	Western-style smoked sausage	Rosemary extract, grape seed extract, green tea polyphenol (0.1%, 0.2%, 0.3%, 0.4%, and 0.5% each)	Green tea polyphenol reduced TBARS by 48.3%, residual nitrite by 68.9%, and N-nitrosamines by 61.3% while improving moisture retention.	[85]
Polyphenol-based antioxidants	Antioxidant	Cooked beef sausage	Pomegranate peel extract, pistachio green hull extract (250 ppm, 500 ppm, 750 ppm, 1000 ppm, and 1250 ppm each)	The 750 ppm pomegranate peel or pistachio extract combined with 60 ppm nitrite maintained TBARS and peroxide values comparable to 120 ppm nitrite control and showed similar sensory scores for color, odor, and taste.	[86]
Polyphenol-based antioxidants	Antioxidant, others (antimicrobial)	Cooked pork sausage	Tomato pomace extract (0.150 µL/g), peppermint essential oil (0.15 µL/g), tomato pomace extract (0.075 µL/g) + peppermint essential oil (0.075 µL/g)	The combination of tomato pomace extract and peppermint essential oil at 0.075 μL/g each with 50 mg nitrite minimized residual nitrite (1.01 mg/kg), lowered TBARS (0.38 mg MDA/kg), and reduced microbial counts.	[87]
Polyphenol-based antioxidants	Antioxidant	Dry fermented sausage	*Juniperus communis* L. essential oil (0.01 µL/g, 0.05 µL/g, and 0.10 µL/g)	At 0.05–0.10 μL/g, TBARS were reduced to 0.14 mg MDA/kg compared to 0.20 mg MDA/kg in the control, while color and microbial quality were preserved.	[88]
Polyphenol-based antioxidants	Antioxidant, others (antimicrobial)	Dry fermented sausage	Sage essential oil (0.01 µL/g, 0.05 µL/g, and 0.10 µL/g)	TBARS values were reduced to 0.12–0.15 mg MDA/kg, and oxidative stability improved during storage; total plate counts remained below 6 log CFU/g throughout 225 days of storage.	[14]
Polyphenol-based antioxidants	Antioxidant	Cured pork sausage	Theaflavins, tea polyphenols (0.03% each)	Theaflavins and tea polyphenols increased a* values and nitrosyl pigment levels, reduced residual nitrite and metmyoglobin, and theaflavins decreased total N-nitrosamines by 30.27% after 28 days.	[29]
Polyphenol-based antioxidants	Antioxidant	Canned pork	Blackcurrant leaf extract (50 mg/kg, 100 mg/kg, and 150 mg/kg)	At 150 mg/kg, TBARS was reduced to 0.029 mg MDA/kg after 180 days, nitrosyl hemochrome reached ~22.0 ppm, and N-nitrosamines were undetectable.	[89]
Polyphenol-based antioxidants	Antioxidant	Emulsion-type pork sausage	Yellow paprika powder (1%, 2%, and 3%)	TBARS were significantly reduced at 2–3% addition levels, while 3% improved water-holding capacity (WHC) and volatile basic nitrogen for 3 weeks storage.	[90]
Polyphenol-based antioxidants	Antioxidant	Cooked sausage	Rosemary extract (0.25%), acerola extract (0.25%), tocopherol mixture (0.05%)	All treatments reduced TBARS and mesophilic bacteria over 23 days; rosemary showed the greatest TBARS inhibition, and all antioxidants reduced redness.	[30]
Polyphenol-based antioxidants	Antioxidant, others (N-nitrosamine inhibition)	Ripened sausage	Olive leaf extract (500 ppm and 1000 ppm)	The 1000 ppm olive leaf extract reduced lipid and protein oxidation and decreased N-nitrosamine formation when combined with reduced nitrite/nitrate levels.	[91]
Combination systems	Curing accelerator, antioxidant, others (antimicrobial)	Spanish chorizo	*Citrus sinensis* L. extract (0.02%), rosemary extract (0.02%), acerola extract (0.01%), lettuce extract (0.30%) + arugula extract (0.15%) + watercress extract (0.15%), spinach extract (0.30%) + celery extract (0.30%), chard extract (0.30%) + beet extract (0.30%)	Combined use reduced hexanal and nonanal levels, improved shelf life, and inhibited *Clostridium perfringens* growth.	[68]
Combination systems	Curing accelerator, antioxidant	Cooked ground pork product	Acerola juice powder (0.1% and 0.2%) + white kimchi powder (0.2% and 0.4%)	A combination of 0.2% white kimchi powder and 0.2% acerola juice powder resulted in the lowest residual nitrite (0.92 ppm), highest nitrosyl hemochrome (41.83 ppm), and a curing efficiency of 79.6%.	[28]
Combination systems	Curing accelerator, antioxidant	Cured pork sausage	Green tea extract powder (0.05% and 0.10%), rosemary extract powder (0.05% and 0.10%), green tea extract powder (0.05%) + rosemary extract powder (0.05%)	Treatments with 0.10% extracts reduced TBARS (0.129–0.130 mg MDA/kg) while maintaining curing efficiency and nitrosyl hemochrome content similar to the control.	[17]
Combination systems	Curing accelerator	Fried beef meatball	Celery powder (0.4–1.2%), Chinese cabbage powder (0.4–1.2%), cranberry powder (0.6–1.4%)	Chinese cabbage powder at 1.0% reduced residual nitrite (13.97 mg/kg), and cranberry powder at 1.0% improved redness (a* = 13.34 vs. 12.64 in nitrite control).	[92]
Combination systems	Curing accelerator, antioxidant	Heat-treated fermented sausage	Pre-converted arugula extract (150 ppm nitrite equivalent), barberry extract (200 ppm gallic acid equivalent)	Pre-converted arugula extract and barberry extract achieved a curing efficiency of 83.5%, exceeding that of nitrite control (67.1%), while barberry extract also lowered TBARS, reduced carbonyl levels, and maintained sulfhydryl group stability.	[73]

**Table 4:** Natural phosphate replacement strategies for clean-label meat products.

Replacement Strategies	Meat Products	Natural Sources and Levels	Major Results	References
Hydrocolloid-based replacer	Low-fat Bologna sausage	Chia seed mucilage gel and chia seed mucilage powder (2% and 4% each)	The 2% chia seed mucilage gel improved emulsion stability and texture in 50% fat-reduced, phosphate-free sausage, with sensory traits similar to the control except for color.	[96]
Hydrocolloid-based replacer	Frankfurter	Micronized cold-pressed sesame seed cake (3–7%)	The 4% micronized cold-pressed sesame seed cake reduced cooking loss by 4.48% and improved emulsion stability, texture, and microstructure; overall acceptability was comparable to the control.	[97]
Protein-based replacer	Chicken meat	Bovine skin gelatin hydrolysate (BSGH1: 6.57%; BSGH2: 13.14%; BSGH3: 26.28% up to 5%)	The 3% BSGH3 addition reduced cooking loss by 70%, matching the performance of 3% sodium tripolyphosphate (STPP), with no significant difference in water-holding capacity (WHC).	[98]
Protein-based replacer	Emulsified cooked sausage	Blood plasma, soy, egg white, pea, potato, gelatin, whey (2% each)	Blood plasma and soy achieved cooking loss and texture similar to phosphate-treated samples, while egg white and pea showed partial functionality.	[99]
Protein-based replacer	Emulsion-type pork sausage	Myofibrillar protein (2%)	Myofibrillar protein improved pH, cooking yield, emulsion stability, and textural properties comparable to phosphate control.	[112]
Protein-based replacer	Brined pork loin	Myofibrillar protein grafted at high concentration (GMP-H, 0.6%) and myofibrillar protein grafted at low concentration (GMP-L, 0.6%)	GMP-H enhanced WHC, tenderness, and thermal stability comparable to phosphate treatment and improved protein interactions.	[113]
Protein-based replacer	Pork meat batter	Chickpea protein isolate (0.5–2.0%)	Addition of 1.5–2.0% chickpea protein isolate significantly reduced cooking loss and enhanced emulsion stability and texture in phosphate-reduced formulations.	[100]
Natural calcium-based replacer	Cooked ground pork products	Oyster shell calcium (0.2%, 0.3%, and 0.5%) + eggshell calcium (0.2%, 0.3%, and 0.5%)	Combined use of 0.2% oyster shell and 0.3% eggshell calcium improved cooking yield, WHC, and texture, comparable to 0.3% phosphate control.	[109]
Natural calcium-based replacer	Ground pork products	Eggshell, oyster shell, marine algae, and milk calcium (0.5% each)	The 0.5% eggshell calcium powder improved the WHC, a*, and pH, but all natural calcium powders resulted in lower textural properties compared to 0.3% phosphate control.	[101]
Natural calcium-based replacer	Ground pork products	Oyster shell calcium, citrus fiber, dried plum powder (0.5% each)	The 0.5% oyster shell calcium maintained cooking yield and lipid separation similar to phosphate; 0.5% citrus fiber and dried plum powder reduced cooking yield.	[13]
Natural calcium-based replacer	Pork patty	Oyster shell powder (0.3% and 0.6%) + Chinese cabbage or radish powder (0.4%)	The 0.3% oyster shell powder reduced cooking loss and shear force, but the 0.6% oyster shell powder impaired curing efficiency.	[102]
Dietary fiber-based replacer	Bologna sausage	Bamboo fiber (2.5% and 5.0%)	The 5.0% bamboo fiber improved emulsion stability, hardness, and chewiness compared to the phosphate control.	[103]
Dietary fiber-based replacer	Deli-style turkey breast	Citrus fiber (0.25% and 0.50%)	Citrus fiber addition did not significantly improve cooking yield or textural properties compared to phosphate-free or STPP-treated controls.	[114]
Dietary fiber-based replacer	Bologna sausage	Citrus fiber (0.5%, 0.75%, and 1.0%)	Citrus fiber at 0.75% maintained yield, whereas 1.0% addition decreased cohesiveness and resilience compared to STPP control.	[115]
Dietary fiber-based replacer	Frankfurter	Soybean husk dietary fiber (1.0–1.5%)	The 1.5% soybean husk dietary fiber enhanced WHC and texture while reducing fat and sodium content.	[104]
Dietary fiber-based replacer	Cooked sausage	Freeze-dried Brussels sprouts, Red Kuri squash, and sweet corn powders (1.0–4.0%)	All treatments improved the WHC (up to 18.4% weight gain); Brussels sprouts matched phosphate-treated samples in texture, and sweet corn showed comparable sensory acceptance.	[15]
Dietary fiber-based replacer	Frankfurter	Seaweed dietary fiber (0.25–1.25%)	The 1.0% seaweed dietary fiber preserved cooking yield and texture but increased Δ*E* and slightly decreased flavor intensity.	[105]
Dietary fiber-based replacer	Emulsified mortadella	Flaxseed cake (1.5% and 3.0%)	The 3.0% flaxseed cake maintained emulsion stability and sensory properties comparable to 0.5% phosphate control, although thiobarbituric acid reactive substances (TBARS) values slightly increased.	[116]
Mushroom-based replacer	Emulsion-type sausage	Winter mushroom powder (0.5%, 1.0%, 1.5%, and 2.0%)	Winter mushroom powder above 1.0% increased pH, reduced jelly and fat exudation by 75% compared to 0% group, lowered TBARS levels more than phosphate control, and maintained acceptable sensory quality up to 1.5%.	[107]
Mushroom-based replacer	Canned ground ham	Plasma-treated winter mushroom powder (1%)	Plasma-treated winter mushroom powder increased redness and improved fat and water loss and TBARS without synthetic nitrite or phosphate control, although it was less effective for phosphate functionality.	[110]
Mushroom-based replacer	Beef patty	Freeze-dried and oven-dried winter mushroom powders (1% each)	Freeze-dried winter mushroom powder reduced cooking loss, while oven-dried powder reduced TBARS; each offered complementary benefits for moisture or oxidative stability.	[117]
Mushroom-based replacer	Emulsion-type pork sausage	Oyster mushroom powder (1% and 2%)	The 2% oyster mushroom powder improved the WHC (74.06%) and reduced cooking loss (2.45%) compared to 0.2% phosphate control (3.38%), although slightly lower hardness was observed.	[106]
Combination system	Ground pork	Natural calcium mixture (0.2% oyster shell calcium and 0.3% eggshell calcium powder) + binding agents (egg white, whey, soy, carrageenan, collagen, 0.25% each)	Natural calcium mixture along with whey protein concentrate or collagen powder enhanced texture; composite systems were comparable to phosphate control.	[12]
Combination system	Restructured turkey steak	Eggshell calcium powder (0.25% and 0.50%) + low-methoxyl pectin (0.25%, powder or gel)	Eggshell calcium powder combined with low-methoxyl pectin gel maintained WHC, with slight redness reduction but high acceptability score.	[108]
Combination system	Low-sodium mortadella	Bamboo fiber (2.5%), isolated pea protein (1.0%), mushroom powder (1.0%)	Combination system enhanced emulsion stability and maintained similar texture; mushroom powder improved flavor but increased lipid oxidation and yellow discoloration.	[32]

## Data Availability

No new data were created or analyzed in this study. Data sharing is not applicable to this article.

## Abbreviations

The following abbreviations are used in this manuscript:

WHC	Water-holding capacity
EU	European Union
LAB	Lactic acid bacteria
TBARS	Thiobarbituric acid reactive substances
MDA	Malondialdehyde
CEP	Citrus peel extract powder
BSGH	Bovine skin gelatin hydrolysate
STPP	Sodium tripolyphosphate
GMP-H	Myofibrillar protein grafted at high concentration
GMP-L	Myofibrillar protein grafted at low concentration
SDF	Seaweed dietary fiber
CPI	Chickpea protein isolate
ESC	Eggshell calcium
OSC	Oyster shell calcium
SHDF	Soybean husk dietary fiber
FDP	Freeze-dried winter mushroom powder
ODP	Oven-dried winter mushroom powder
PWMP	Plasma-treated winter mushroom powder
WPC	Whey protein concentrate
HPP	High-pressure processing
US	Ultrasound
CP	Cold plasma
CSS	Ultrasound-treated coffer silver
CP-PBS	Cold plasma-treated phosphate solution
